# Telephone-Cardiopulmonary Resuscitation Guided by a Telecommunicator: Design of a Guiding Algorithm for Telecommunicators

**DOI:** 10.3390/jcm12185884

**Published:** 2023-09-10

**Authors:** Tamara Rafaela Yacobis-Cervantes, Juan Antonio García-Méndez, César Leal-Costa, María Ángeles Castaño-Molina, María Suárez-Cortés, José Luis Díaz-Agea

**Affiliations:** 1 Faculty of Nursing, Cartagena Campus, Catholic University of Murcia, 30310 Cartagena, Spain; tryacobis@ucam.edu (T.R.Y.-C.); jagmendez@ucam.edu (J.A.G.-M.); 2 Faculty of Nursing, Campus de Ciencias de la Salud, University of Murcia, 30120 Murcia, Spain; angeles.castano@um.es (M.Á.C.-M.); maria.suarez@um.es (M.S.-C.); agea@um.es (J.L.D.-A.)

**Keywords:** cardiopulmonary resuscitation, cardiac arrest, survival, out-of-hospital cardiac arrest (OHCA), telephone-cardiopulmonary resuscitation

## Abstract

Background: Out-of-hospital cardiac arrest is considered a global problem. In the last few years, there has been a growing interest in telephone-cardiopulmonary resuscitation guided by a telecommunicator. Indeed, several studies have demonstrated that it increases the chances of survival rate. This study focuses on the key points the operator should follow when performing telephone-cardiopulmonary resuscitation. The main objective of this paper is to design an algorithm to improve the telephone-cardiopulmonary resuscitation response protocol. Methods: The available evidence and the areas of uncertainty that have not been previously mentioned in the literature are discussed. All the information has been analyzed by two discussion groups. Later, a consensus was reached among all members. Finally, a response algorithm was designed and implemented in clinical simulation. Results: All the witnesses were able to recognize the OHCA, call for emergency assistance, follow all the operator’s instructions, move the victim, and place their hands in the correct position to perform CPR. Discussion: The results of the pilot study provide us a basis for further experimental studies using randomization and experimental and control groups. Conclusions: No standardized recommendations exist for the operator to perform telephone-guided CPR. For this reason, a response algorithm was designed.

## 1. Introduction

Out-of-hospital cardiac arrest (OHCA) is considered a global problem [1] and represents one of the leading causes of death worldwide. According to recent data, North America has the highest rates, followed by Europe, Asia, and Australia. On the other hand, the survival rate is highest in Australia, followed by North America, Europe, and Asia [2].

According to data from the European Resuscitation Council (ERC) [2], cardiac arrest is one of the leading causes of death in Europe. Around 20–25% of the victims present ventricular fibrillation, although when the rhythm is analyzed by the emergency services, it has usually changed to asystole. When the ventricular fibrillation rhythm is identified immediately after collapse by an automatic external defibrillator (AED) and a bystander performs cardiopulmonary resuscitation (CPR) while the victim is in ventricular fibrillation, the survival rate is higher [3]. 

In the last few years, there has been a growing interest in telephone-cardiopulmonary resuscitation (CPR) guided by a telecommunicator, also called telephone-guided CPR. Indeed, several studies have demonstrated that it increases the chances of CPR success [2,3,4,5,6].

The average time from the call to the emergency services until the emergency services arrive is between 5 to 8 min, and the average time in which the first cardiac defibrillation is made is around 8 to 11 min. Immediate initiation of CPR can double or even quadruple survival after cardiac arrest [2]. This highlights the importance of starting CPR as soon as possible. In the field of telephone-guided CPR, operators play an important role in the early recognition of cardiac arrest. They play an essential role in order to guide bystander rescuers in how to perform high-quality CPR. They must be aware of the presence of an AED nearby and report it if so. To recognize CPR, they must encourage the person to find out if the victim responds or presents seizures as they might precede cardiac arrest. In the case the victim does not respond and does not breathe, telephone-guided CPR should begin. According to the ERC guidelines, bystanders should perform compression-only CPR on an adult until the arrival of an AED, better-trained rescuers, or emergency systems (EMS) or until the victim begins to move.

Currently, the protocols for telephone-cardiopulmonary resuscitation guided by a telecommunicator are different in each country. There are no unified criteria on which these protocols are based, so most of them just follow the high-quality CPR recommendations by ERC [2] and AHA [4].

There is a difference between ERC’s [2] and AHA’s [4] guides on this issue. In its latest guide, the AHA [4] divides the adult chain of survival into two different chains: in-hospital cardiac arrest (IHCA) and out-of-hospital cardiac arrest (OHCA), including lay rescuers, also called witnesses, as the first link in the chain of survival of out-of-hospital cardiac arrest. However, there is a consensus between the two guidelines [2,4] about five criteria of high-quality CPR, which telecommunicators should consider when guiding CPR: perform chest compressions in the center of the chest at a rate of 100 to 120 compressions per minute; compress a minimum depth of 5 cm, but not more than 6 cm in the average adult. In children, 5 cm and in infants, 4 cm; allow the chest to fully re-expand after each compression and do not remain supported on the victim’s chest; if the bystander who calls emergencies reports seizures, the telecommunicator should have a high suspicion of cardiac arrest, even if the victim has a prior history of epilepsy; CPR should be initiated in the presence of agonal breathing (gasping).

Another important issue is that there is no standard training for telecommunicators, prompting the need for further consideration. The role of telephone-guided CPR operators requires specific training in order to provide clear and effective instructions within the context of a stressful situation. Training in non-technical skills, such as communication and leadership, should be incorporated into life support courses [2]. ERC [2] and AHA [4] mention it is a priority to unify existing protocols, specifically to train telecommunicators, as well as establish periodic evaluations of the quality of telephone CPR assistance [7].

Thus, this study focuses on the key points that the operator should follow as a guide when performing telephone-cardiopulmonary resuscitation. The available evidence regarding this problem is discussed in this article, as well as the areas of uncertainty that we have considered interesting and that have not been previously mentioned in the literature. 

The aim of this project was to propose a realistic and valid protocol design in order to improve the telephone-cardiopulmonary resuscitation response protocol. For that purpose, we have evaluated the currently available evidence as well as identified the areas of uncertainty that have not been previously discussed. All the information has been analyzed by two discussion groups to obtain conclusions that can contribute to considering new ideas and propose points for improvement. In addition, this algorithm has been implemented in clinical simulation as a pilot study to ensure its effectiveness so it could be applied to the general population in future research.

## 2. Materials and Methods

The primary objective was to design an algorithm to improve the telephone-cardiopulmonary resuscitation response protocol. The secondary objectives were to describe the current scientific evidence regarding cardiopulmonary resuscitation and telephone-cardiopulmonary resuscitation guided by a telecommunicator, to identify areas of uncertainty, to analyze two discussion groups regarding the available scientific evidence and the areas of uncertainty, and finally, to facilitate a pilot study implementing the algorithm by clinical simulation.

Five phases were established for this project. All of them are described in depth below.

### 2.1. Literature Review: Scoping Review 

First, a search for the available scientific evidence on cardiopulmonary resuscitation and telephone-cardiopulmonary resuscitation guided by a telecommunicator was carried out, followed by a critical review of the articles found. A scoping review was accomplished instead of a systematic review due to the methodology of the articles found to be quite diverse to conduct a systematic review. Additionally, scoping reviews were conducted when the purposes of the study were to identify knowledge gaps (also called areas of uncertainty), to scope a body of literature, to clarify concepts, and to explore different research perspectives. 

The databases used were Pubmed and Cochrane. In addition, Google’s academic web browser was also used to search for worldwide guides and related protocols. The keywords that were used are as follows:

Indexed in the MeSH browser: cardiopulmonary resuscitation; cardiac arrest; survival; out-of-hospital cardiac arrest.

Not indexed in the MeSH browser due to being a recent term: telephone cardiopulmonary resuscitation. Additionally, found as CPR guided by phone; telephone-cardiopulmonary resuscitation guided by a telecommunicator; guided or assisted telephone CPR; CPR performed by a layperson.

Inclusion criteria: observational, experimental, and quasi-experimental studies.

**Figure 1:**
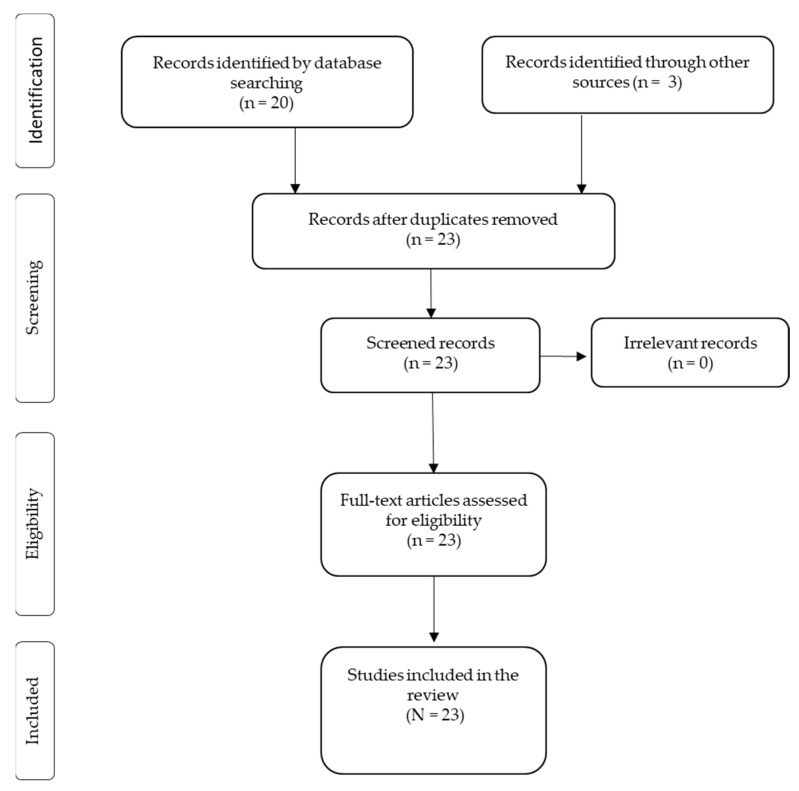
Study flow chart.

Exclusion criteria: studies considered not relevant to the aim of this research and duplicate articles (Figure 1).

### 2.2. Identifying Areas of Uncertainty

Secondly, after establishing a framework of reference regarding the available scientific evidence provided by the articles, we considered the areas of uncertainty cited, as well as some others that we have estimated essential to increase the survival rate. The five areas identified are mentioned in the results.

### 2.3. Discussion Groups 

Thirdly, all the information was analyzed by two discussion groups made up of four people each. Both groups included the following healthcare professionals: one expert psychologist in communication to deal with the bystander approach; one advanced life support provider to provide a practical point of view; one advanced life support instructor to ensure the theoretical point of view; one telephone-guided CPR operator nurse with professional experience to offer a global point of view since they have real experience with the studied subject. The two groups were led by two of the nurse authors of this study, who wrote down and classified the conclusions according to the five areas of uncertainty by each group. Later, the general conclusions were discussed collectively, and a group consensus was reached among all members. These conclusions were elaborated in more detail and individually presented to each of the experts, who confirmed their validity and gave their individual approval.

The experts participated in the discussion groups voluntarily and were informed regarding the purpose of the study.

The information obtained from the discussion groups was transcribed and subjected to a classified content analysis and then presented by the classified areas or dimensions.

### 2.4. Design of a Guiding Algorithm for Telecommunicators

**Figure 2:**
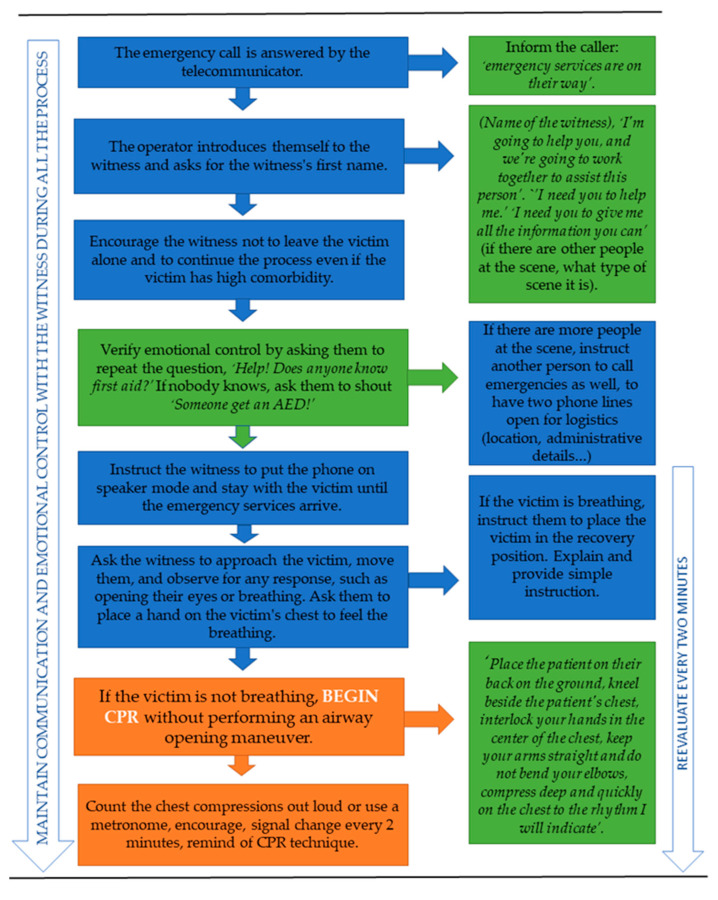
Guiding algorithm for telecommunicators.

We used a new approach for telephone-cardiopulmonary resuscitation guided by a telecommunicator. Based on these conclusions, a response algorithm was developed, unifying the current scientific evidence and summarizing it in a clear, concise, and visual manner that is easy to implement by telecommunicators, indicating the phases and exact words that were consensus among the discussion groups as the most suitable. Additionally, we used different colors for each phase (Figure 2).

### 2.5. Implementing the Algorithm by Clinical Simulation

As a pilot study, the algorithm was tested through a 5 min clinical simulation scenario involving 10 non-healthcare volunteer participants selected by convenience sample. They performed a telephone-guided CPR following the algorithm under the guidance of an expert emergency nurse as a telecommunicator. During the scenarios, the five areas of uncertainty were considered and observed. In addition, six quantitative variables were measured to test the algorithm (Table 1): average compression depth, average compression rate, percentage of compressions with appropriate frequency, percentage of compressions with full recoil, percentage of compressions with sufficient depth, and percentage of compressions with correct hand placement. Data on the variables were extracted from the high-fidelity dummy software (Resusci Anne QCPR RQI 2021. Laerdal Medical Tanke Svilandsgate 30, 4002 Stavanger, Norway).

The simulator used was “Resusci Anne QCPR Full Body” by Laerdal (Laerdal Medical Tanke Svilandsgate 30, 4002 Stavanger, Norway).

## 3. Results

### 3.1. Literature Review: Scoping Review

Despite the absence of a unified protocol regarding CPR guided by a telecommunicator, 22 of the reviewed articles [2,3,4,5,6,7,8,9,10,11,12,13,14,15,16,17,18,19] concur that the following steps should always be considered:

- The telecommunicator should ask whether or not the victim is breathing normally and instruct them to start CPR if they are not breathing or have agonal breathing.
- If the victim is convulsing, PCR should be suspected, even if the victim has a prior history of seizures.
- The telecommunicator must know the availability of a nearby AED based on the location of the PCR and notify the witness. Additionally, if there are other witnesses, they should be encouraged to find a nearby DEA.
- If the witness has no experience performing CPR, it should be indicated that only chest compressions be performed unless it is a child, in which case rescue breaths will be performed following the 30:2 frequency.
- The pauses between compressions and ventilations should be less than 10 s, and insufflation for one second.
- The telecommunicator must clearly indicate that chest compressions are performed in the center of the chest as soon as possible, with a frequency of 100–120 compressions per minute. To achieve it, the compressions must be counted out loud, or a metronome can be used.
- The telecommunicator should encourage deep compressions to ensure a minimum depth of 5 cm, but no more than 6 cm in adults. In children, 5 cm and in infants, 4 cm. You should also keep reminding the witness that you must allow the chest to re-expand for the compressions to be effective.
- If there is more than one witness, they should alternate to performing CPR every two minutes or as needed to ensure high-quality CPR until emergency services arrive.

### 3.2. Identifying Areas of Uncertainty 

The five areas of uncertainty that have been identified are the following:

- How the telecommunicator can facilitate effective communication and manage the emotional shock of the witness or lay rescuer in a stressful situation.
- Strategies to enhance the telecommunicator’s ability to accurately identify cardiac arrest over the phone.
- Methods to enhance the depth of compressions performed by the lay rescuer.
- Steps to assist the lay rescuer when unable to move the victim.
- How to identify another person to help the witness (lay rescuer) in case the caller is unable to do so, or to take turns every two minutes or as needed, ensuring high-quality CPR.

### 3.3. Discussion Groups 

The general conclusions achieved classified by dimensions were the following: 

#### 3.3.1. How the Telecommunicator Can Facilitate Effective Communication and Manage the Emotional Shock of the Witness or Lay Rescuer in a Stressful Situation

To achieve emotional control of the calling witness, it is important for the telecommunicator to have proper training. Several points are important to consider, which will be enumerated below in chronological order. The telecommunicator must introduce themselves to the calling witness and ask for the witness’s first name, using it to address them at the beginning of each sentence or when necessary to regain their attention. Inquire about the relationship between the caller and the affected person to better understand the situation and adapt accordingly. Assure the witness that emergency services have been alerted and are on their way as soon as possible, reiterating as necessary or whenever the witness asks. The collaboration of the witness is vital for the success of CPR. To ensure this collaboration, the operator can use phrases such as *‘I am going to help you, and we will work together to assist this person’*, *‘I need your help’*, and *’I need you to provide all the information you can’*. This aims to make the witness an active participant, absolving them of blame or responsibility if CPR is not successful. If the witness is unwilling to cooperate, efforts should be made to encourage them. Generally, the telecommunicator should adapt their vocabulary to the socio-cultural level of the witness and avoid using technical terms. They should use understandable language with a calm tone and appropriate speed, conveying reassurance and confidence. The communication style should be direct, providing simple instructions to guide the witness performing CPR. It is important to listen to the witness without interrupting unless redirection is needed.

In addition, the operator should be able to identify and recognize the witness’s emotions and adapt to them throughout the call to achieve better emotional control. Throughout the call, the operator should use various emotional control techniques, such as positive reinforcement, feedback, maintaining continuous verbal contact, and not engaging in provocations if initiated by the witness. Encourage the witness not to leave the victim alone and to continue the process even if the victim has high comorbidity. Once the operator deems that emotional control has been achieved, ask if there are more people around who can help. To assess if the operator has emotional control over the witness, they can ask them to shout the following question if they are surrounded by more people: *‘Help! Does anyone know first aid?’*. If no one knows, instruct them to shout, *‘Someone, get an AED!’*. If there are more people, ask the witness to ask them for help, have them approach the victim, and begin guiding CPR using the available resources in each situation.

#### 3.3.2. Strategies to Enhance the Telecommunicator’s Ability to Accurately Identify Cardiac Arrest over the Phone

The telecommunicator should instruct the witness to stay with the victim until the medical services arrive. To detect cardiac arrest, the telecommunicator should instruct the witness to approach the victim, shake them gently, and see if there is any response, if they open their eyes, and if they are breathing. To determine if the victim is breathing or not if the witness is unsure, the telecommunicator will instruct the witness to place a hand on the victim’s chest. If the victim is responsive or unresponsive but breathing, they should instruct the witness to place the victim in the recovery position. The telecommunicator should explain what this entails and provide simple instructions for moving the victim. If the victim is not breathing, the operator should instruct the witness to begin CPR without performing an airway opening maneuver to avoid wasting time.

#### 3.3.3. Methods to Enhance the Depth of Compressions Performed by the Lay Rescuer

The telecommunicator should guide the witness in performing CPR by exclusively delivering chest compressions. Explain the CPR technique in a simple manner: “Place the patient on their back on the floor, kneel beside the patient’s chest, interlock your hands in the center of the patient’s chest, keep your arms straight, and avoid bending your elbows, compress forcefully and rapidly on the chest to the rhythm I will indicate.” The operator should verbally announce the number of compressions to guide the witness. This should periodically remind the witness of the CPR technique. They should ask if their arms are fully extended and if they are compressing forcefully and rapidly whilst also allowing for full chest recoil. 

They should remind the witness to continue their own breathing to avoid becoming tired. They should encourage the witness throughout the call and employ emotional control techniques. They should instruct the witness that if there is another person available, they should switch whenever they become fatigued or as directed by the operator (the telecommunicator will count every two minutes to signal the switch). Minimize interruptions during transitions as much as possible.

#### 3.3.4. Steps to Assist the Lay Rescuer When Unable to Move the Victim

The operator should instruct the witness to place the victim on their back on a hard surface, such as the ground, before beginning CPR. It should advise the witness to move the victim from an inaccessible location or a small, uncomfortable space for performing CPR, such as a bathroom. To move the victim, if they are on a bed, the operator should instruct the witness to move them to the floor by pulling the sheet, taking care to avoid hitting the head. If the victim is seated or in a position where the witness has sufficient strength to move them, they can be instructed to place their arms under the victim’s armpits and pull to position them on the floor face up.

#### 3.3.5. How to Identify Another Person to Help the Witness or Lay Rescuer in Case the Caller Is Unable to Do so, or to Take Turns Every Two Minutes or as Needed, Ensuring High-Quality CPR

If the witness is surrounded by more people, the telecommunicator should encourage the witness to seek another person from their surroundings by shouting the question, *‘Help! Does anyone know how to perform first aid?’*. If no one knows, instruct them to shout, *‘Someone, get an AED!’*. If there are more people, instruct the witness to ask for their help, have them approach the victim, and begin guiding CPR with the available resources in each situation. If there are additional individuals present, encourage another person to call emergencies again to have two phone lines open for logistical purposes (location information, administrative details). 

The telecommunicator should use all available resources in each situation, for which they would have already asked the witness to provide as much information as possible about the scenario (number of people present, address, shopping center, public area, etc.). If there are multiple people, involve everyone in CPR, indicating that they should switch when fatigued or every two minutes, as per CPR quality standards. If the cardiac arrest occurs in a secure location like a shopping center, instruct the witness to have someone notify security to locate an AED or assist with CPR. If the OHCA occurs in a nursing home and the witness is alone, instruct them to open the door of the building before performing CPR, allowing the medical services to enter the premises without interrupting CPR to open the door upon their arrival. The same is true if the witness is alone at home. 

#### 3.3.6. Design of a Guiding Algorithm for Telecommunicators

This algorithm summarizes the essence of the conclusions and agreements of the focus groups on how to handle an out-of-hospital cardiorespiratory arrest call (Figure 2).

In the algorithm, the blue squares indicate the actions that the operator must perform, and the green squares indicate the orders with the exact words the operator must provide to the witness. The orange squares show the start of the witness performing CPR on the victim.

#### 3.3.7. Implementing the Algorithm by Clinical Simulation

Table 1 displays the quantitative data obtained during the implementation of the algorithm in clinical simulation scenarios.

Even though the witnesses were not of the same physical strength, age, and gender, they were all able to recognize the OHCA, call for emergency assistance, follow all the operator’s instructions, move the victim to the supine position, and place their hands in the correct position.

## 4. Discussion

As mentioned before, in the literature review, it was found that there is currently no unified protocol for operators performing telephone-guided CPR. Instead, each country has its own guidelines based on the principles of high-quality CPR.

Studies on telephone-guided CPR have been conducted worldwide. All of them provide different scientific evidence supporting its effectiveness, and, at the same time, they all agree on the need for further research to understand current strengths and weaknesses and to standardize and implement a protocol [1,2,3,4,5,6,7,8,9,10,11,12,13,14,15,16,17,18,19,20,21].

Some research findings are similar in certain aspects: several studies agree that it is vital for the operator to count compressions to encourage the person performing CPR. This has been shown to improve the number of compressions but not the depth [8,9]. A study based on communication demonstrated that communication between the operator and the rescuer was easier when it was clear, concise, and had some form of short feedback. However, the same results were obtained regarding actions performed by the rescuer in both the effective communication group and the ineffective communication group. This suggests that there are other influencing factors besides communication between the operator and the rescuer [10].

Another research conducted in France clearly identified three predictive factors for success in telephone-guided CPR: effective use of communication tools, the ability to identify a suitable person to perform CPR, and proper positioning of the victim prior to initiating CPR. The absence of a familial relationship between the rescuer and the victim was also identified as one of the reasons influencing the immediate start of telephone-guided CPR. Therefore, the study suggests that further research on these factors is important. Other factors cited as difficulties when performing CPR included the rescuer’s inability to move the victim, the inability to perform CPR due to emotional shock, and the operator’s inability to detect cardiac arrest [11].

In its latest guidelines, the American Heart Association (AHA) [4] divides the adult chain of survival into two different chains: in-hospital cardiac arrest (IHCA) and out-of-hospital cardiac arrest (OHCA), including lay rescuers or witnesses as the first link in the chain of survival. The European Resuscitation Council (ERC) [2] does not make this distinction, but like the AHA, it includes recommendations for telephone-guided CPR in its latest guidelines. Furthermore, one notable difference should be considered, which is that the AHA [4] recommends intramuscular or intranasal administration of naloxone if the lay rescuer suspects opioid substance addiction in the victim and is a trained rescuer, whereas the ERC [2] does not include this recommendation.

This study involved a literature search, the establishment of a protocol, and a pilot test using clinical simulation. The algorithm could be the basis for improved out-of-hospital care of cardiorespiratory arrests attended by bystanders calling emergency services and answered by telephone operators. 

As this is a pilot study, the results are incomplete and refer to an insufficient sample as has been conducted at a local level. Given that our findings implementing the algorithm are based on a limited number of people, we suggest that a large-scale study should be conducted, and implementing it in real practice is of vital importance for future research. To prove that this algorithm is adequate and valid, it should be tested in an experimental study with a control group. The results of the pilot study provide us a basis for further experimental studies using randomization and experimental and control groups.

## 5. Conclusions

Currently, there are no standardized recommendations for the operator to perform telephone-guided CPR. For this reason, a response algorithm was developed in this study. The algorithm is based on two previous focus groups in which the main steps for guiding telephone CPR were identified.

To conclude, it is a priority to unify existing protocols, specifically to train telecommunicators, as well as establish periodic evaluations of the quality of telephone-cardiopulmonary resuscitation guided by a telecommunicator to increase the survival rate of OHCA. 

## Figures and Tables

**Table 1:** Results of clinical simulation scenarios.

	Average Compression Depth	Average Compression Rate	% Compressions with Appropriate Frequency	% of Compressions with Full Recoil	% of Compressions with Sufficient Depth	% of Compressions with Correct Hand Placement
Witness 1	4.5 cm	110/min	98%	46%	12%	95%
Witness 2	5.8 cm	110/min	98%	2%	95%	100%
Witness 3	4.6 cm	87/min	1%	0%	28%	100%
Witness 4	5.8 cm	110/min	98%	2%	95%	100%
Witness 5	5.6 cm	107/min	87%	42%	93%	100%
Witness 6	4.2 cm	140/min	3%	8%	6%	100%
Witness 7	5.5 cm	78/min	0%	100%	95%	100%
Witness 8	5.6 cm	107/min	87%	42%	93%	100%
Witness 9	4.7 cm	104/min	35%	86%	83%	100%
Witness 10	4.5 cm	110/min	98%	40%	95%	100%

## Data Availability

The data used to support the findings of this study are available from the corresponding author upon request.
